# The lipid flippase Drs2 regulates anterograde transport of Atg9 during autophagy

**DOI:** 10.1080/27694127.2022.2104781

**Published:** 2022-08-09

**Authors:** Franziska Kriegenburg, Wouter Huiting, Fleur van Buuren-Broek, Emma Zwilling, Ralph Hardenberg, Muriel Mari, Claudine Kraft, Fulvio Reggiori

**Affiliations:** a Department of Biomedical Sciences of Cells and Systems, University of Groningen, University medical Centre Groningen, 9713AV Groningen, The Netherlands; b Institute of Biochemistry and Molecular Biology, ZBMZ, Faculty of Medicine, University of Freiburg, 79104, Freiburg, Germany; c Department of Biomedicine, Aarhus University, Ole Worms Allé 4, 8000 Aarhus C, Denmark; d CIBSS - Centre for Integrative Biological Signalling Studies, University of Freiburg, 79104, Freiburg, Germany

**Keywords:** Aminophospholipid, Atg protein, autophagosome, flippase, lipid asymmetry, phagophore, phagophore assembly site

## Abstract

Macroautophagy/autophagy is a conserved catabolic pathway during which cellular material is sequestered within newly formed double-membrane vesicles called autophagosomes and delivered to the lytic compartment of eukaryotic cells for degradation. Autophagosome biogenesis depends on the core autophagy-related (Atg) machinery, and involves a massive supply and remodelling of membranes. To gain insight into the lipid remodelling mechanisms during autophagy, we have systematically investigated whether lipid flippases are required for this pathway in the yeast *Saccharomyces cerevisiae*. We found that the flippase Drs2, which transfers phosphatidylserine and phosphatidylethanolamine from the lumenal to the cytosolic leaflet of the limiting membrane at the trans-Golgi network, is required for normal progression of autophagy. We also show that Drs2 is important for the trafficking of the core Atg protein Atg9. Atg9 is a transmembrane protein important for autophagosome biogenesis and its anterograde transport from its post-Golgi reservoirs to the site of autophagosome formation is severely impaired in the absence of Drs2. Thus, our results identify a novel autophagy player and highlight that membrane asymmetry regulates early autophagy steps.

**Abbreviations:** ABs: autophagic bodies; Atg: autophagy-related; BiFC: bimolecular fluorescence microscopy; Cvt: cytoplasm-to-vacuole targeting; ER: endoplasmic reticulum; P4-ATPases: type IV P-type ATPases; PAS: phagophore assembly site; PE: phosphatidylethanolamine; PS: phosphatidylserine; PtdIns3P: phosphatidylinositol-3-phosphate; TGN: trans-Golgi network; WT: wild type

## Introduction

Macroautophagy (hereafter autophagy) is a catabolic pathway conserved among eukaryotes. It is characterized by the formation of a membranous cisterna, known as the phagophore, which sequesters cellular material while expanding and closing into a double-membrane autophagosome [1]. Autophagosomes subsequently fuse with the lysosome (in mammals) or vacuole (in yeast/plants), in which their cargo is hydrolyzed and the resulting metabolites are recycled to replenish the pool of available metabolites and energy sources [2]. Thus, autophagy not only allows cells to remove dysfunctional and/or superfluous cellular components, but also secures survival during starvation conditions [2]. Depending on the autophagy stimulus, autophagy can be subdivided into nonselective, “bulk” autophagy when the autophagosome content is heterogenous, and selective autophagy, during which specific cytoplasmic structures are exclusively targeted for autophagic degradation by the so-called autophagy receptors [3]. A type of selective autophagy in yeast is the biosynthetic cytoplasm-to-vacuole targeting (Cvt) pathway, which delivers vacuolar enzymes such as Ape1 (aminopeptidase I) into the vacuole [4].

Autophagy can be subdivided into discrete steps: autophagy initiation, phagophore nucleation and elongation, phagophore closure to form an autophagosome, autophagosome maturation and its fusion with the lytic compartment and cargo degradation ,^5^]. The steps underlying autophagosome biogenesis are orchestrated by a core machinery composed by highly conserved autophagy-related (Atg) proteins [1,5]. During autophagy initiation in budding yeast, oligomeric protein heterocomplexes centered around the core kinase Atg1 self-assemble into a supramolecular structure that undergoes phase-separation [1,6], forming the so-called phagophore assembly site (PAS) [7-9]. To this site, which is confined by the vacuole and the endoplasmic reticulum (ER), Atg9-containing and possible COPII-coated vesicles are engaged and they provide at least in part the membranes required for the phagophore formation [1,5]. Further, the class III phosphatidylinositol 3-kinase complex I is recruited to the PAS, where it synthesizes phosphatidylinositol-3-phosphate (PtdIns3P) important for the recruitment of PtdIns3P downstream effectors, like Atg18, Atg2 and components of two ubiquitin-like conjugation systems [1,5]. The latter covalently link the ubiquitin-like protein Atg8 to phosphatidylethanolamine (PE) present in the phagophore membrane. Lipidated Atg8 controls cargo selection and phagophore expansion. Phagophore expansion also depends on a continuous supply of lipids and it has been shown that Atg2, in cooperation with Atg18 and Atg9, transfers lipids from the ER to the phagophore. In particular, Atg2 is key in generating a membrane contact site between the ER and the extremities of the growing phagophore [10] and allows lipids to flow through its tunnel-like protein structure [11-13]. Both Atg18 and the transmembrane protein Atg9 are required to anchor Atg2 onto the phagophore extremities [10]. In addition, Atg9 has scramblase activity *in vitro* and is suggested to distribute incoming lipids from the cytosolic to the luminal leaflet of the phagophore membrane, as these would otherwise create a biophysical imbalance due to an accumulation of phospholipids in the outer lipid bilayer [14,15]. As scramblases facilitate ATP-independent lipid redistribution with limited specificity, this could lead to a reduction in lipid asymmetry within the phagophore membrane that in turn may be important for autophagosome formation. For example, it has been proposed that protein and possibly lipid asymmetrical distribution between the leaflets of the phagophore membrane is crucial to generate and stabilize phagophore curvature [16]. We therefore wondered whether there are additional lipid translocation machineries at the nascent phagophore that may be involved in the generation of lipid asymmetry within the autophagosomal membrane. Interestingly, proteomics approaches have identified lipid flippases such as Drs2, Neo1 or Dnf1 and Dnf2 within the autophagy protein network [17,18] (our own unpublished data).

Lipid flippases are type IV P-type ATPases (P4-ATPases) which translocate phospholipids unidirectionally in an ATP-dependent manner [19,20]. The resulting asymmetry in membrane lipid distribution is involved in signal transduction, apoptosis and membrane trafficking [20-22]. P4-ATPases are large proteins with 10 transmembrane domains, which determine flippase substrate specificity and along which the phospholipid head group is transported across the lipid bilayer [23-27]. Lipid flippases contain a conserved key aspartic residue that undergoes transient phosphorylation during the lipid translocation [20,28]. The translocation activity of the flippases is regulated by their cytosolic N- and C-terminal domains, and/or via phosphorylation [29-32]. Moreover, several flippases rely on the presence of an accessory subunit belonging to the Cdc50-Lem3 protein family, which promotes flippase export from the ER as well as their lipid translocase activity [33-37]. *S. cerevisiae* encodes for five flippases: Dnf1, Dnf2, Dnf3, Drs2 and Neo1 [38]. While *NEO1* is the only essential gene, the combination of *drs2∆ dnf1∆ dnf2∆ dnf3∆* also causes lethality, consistent with the notion that these four flippases are partially redundant [38]. Drs2 is the best characterized P4-ATPase in yeast. It is primarily localized at the trans-Golgi network (TGN) and it mainly flips phosphatidylserine (PS), but also PE, from the inner to the cytosolic leaflet of the limiting membrane of the TGN, thereby initiating membrane curvature and priming vesicle budding [28,39,40].

In this study, we reveal that the P4-ATPase Drs2 is important for autophagy progression in yeast by modulating Atg9 anterograde transport to the PAS.

## Results

### Deletion of the gene encoding the Drs2 lipid flippase affects autophagy

**Figure 1.**
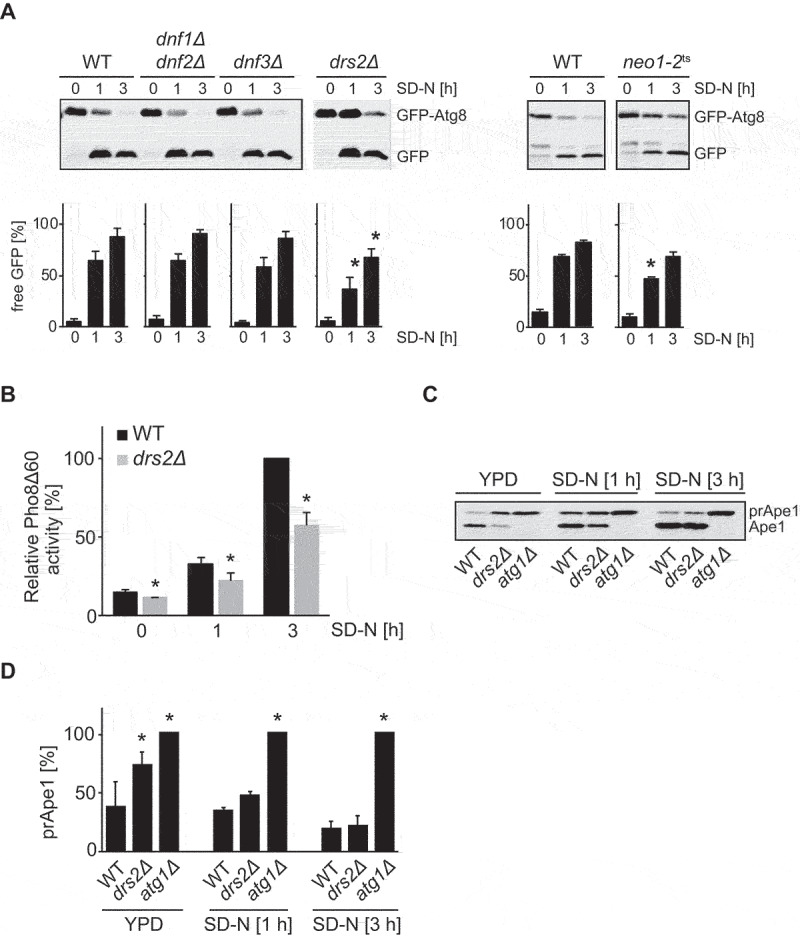
Deletion of the gene encoding the flippase Drs2 in *S. cerevisiae* results in defective autophagy. (**A**) Wild type (WT) (SEY6210) and the indicated flippase mutant strains (TPY026; TPY015; TPY010; ZHY124-34A2B) were transformed with a plasmid expressing GFP-Atg8 under the control of the *CUP1* promoter (pRS416CuprGFP-Atg8), grown in SMD-URA medium at 30°C to late log phase and shifted to SD-N medium to induce autophagy. Cells were harvested after 0, 1 and 3 h, and precipitated in 10% TCA. GFP-Atg8 processing was assessed by western blot using an anti-GFP antibody. To induce the temperature-sensitive phenotype, the *neo1-2^ts^* strain was grown in SMD-URA medium at 24°C and then incubated for 1 h at 37°C before autophagy induction in SD-N medium at 37°C. Cells were collected after 0, 1 and 3 h. The percentage of free GFP in comparison to total GFP, i.e., free GFP plus GFP-Atg8, was quantified and plotted. The bars present the average values from three independent experiments plus standard error (SEM). The asterisks indicate significant differences in the GFP-Atg8 turnover between the flippase mutant strains and the WT at the indicated time point (two-tailed Student’s t-test, p-value < 0.05). (**B**) WT (YTS159) and *drs2Δ* (FBY015) cells carrying the Pho8Δ60 reporter were grown in YPD at 30°C to late log phase and then shifted to SD-N medium for 1 h and 3 h. Cells were collected before and after the incubation in SD-N, lysed and the Pho8Δ60-dependent phosphatase activity was measured. All values were normalized to the Pho8Δ60 activity of the WT. The bars present the average values from three independent experiments plus SD. The asterisks indicate significant differences in bulk autophagy between the *drs2*Δ strain and the WT at the indicated time points (two-tailed Student’s t-test, p-value < 0.05). (**C**) WT (SEY6210), *atg1*Δ (WHY1) and *drs2*Δ (FKY758) strains were grown in YPD at 30°C to late log phase and shifted to SD-N medium. Cells were harvested after 0, 1 and 3 h, and precipitated in 10% TCA. prApe1 processing into Ape1 was examined by western blot using an anti-Ape1 antibody. One representative experiment out of three independent ones is shown. (**D**) The maturation of prApe1 into Ape1 shown in panel C was quantified and the percentages of remaining prApe1 over total Ape1 was plotted. The bars represent the average values from three independent experiments plus SD. The asterisk indicates significant differences in the Cvt pathway between the *drs2*Δ or *atg1*Δ cells, and the WT at the indicated time point (one-way ANOVA, p-value < 0.05 or smaller).

We first tested whether the lack of any of the five *S. cerevisiae* lipid flippases has a general autophagy defect using the GFP-Atg8 processing assay [41]. Because both Dnf1 and Dnf2 function redundantly at the plasma membrane [42], we used a strain lacking both flippases for the assay. In addition, to analyze Neo1, the only essential flippase, we took advantage of the temperature-sensitive mutant *neo1-2* [43]. The wild type (WT) and the lipid flippase mutant strains were transformed with a centromeric plasmid expressing GFP-Atg8 and subsequently grown in rich medium to exponential phase and shifted to nitrogen starvation for 3 h to induce bulk autophagy. While WT and the deletion strains were used at 30°C, the *neo1-2* mutant was grown at the permissive temperature of 24°C and transferred to 37°C for 1 h prior to nitrogen starvation at the same temperature for 3 h. Samples were collected at 0, 1 and 3 h before precipitating proteins with trichloroacetic acid and analyzing them by western blot using GFP antibodies. As depicted in Fig. 1A, increase of free GFP over time and concomitant decrease of GFP-Atg8 in WT reflect the normal progression of autophagy [41]. Processing of GFP-Atg8 in *dnf3∆* and *dnf1∆ dnf2∆* cells was identical to WT indicating that the encoded flippases are not required for autophagy. In contrast, deletion of *DRS2* caused a significant reduction in GFP-Atg8 degradation (Fig. 1A). Inactivation of *NEO1* also had a negative impact on the processing of GFP-Atg8, but the detected difference was significant only at the 1 h time point. As results from our laboratory and others have previously shown the importance of the yeast Golgi functions in autophagy [44-50], we focused on the Golgi-resident flippase Drs2.

To corroborate the autophagy defect of the *drs2∆* mutant, we also took advantage of the Pho8∆60 assay [41]. Pho8∆60, the truncated variant of the vacuolar phosphatase Pho8, can only be transported from the cytosol into the vacuole by bulk autophagy, where it is activated by proteolytic cleavage. After 3 h of nitrogen starvation, a 40 % reduction in Pho8∆60 activity was observed in *drs2∆* cells compared to WT (Fig. 1B).

Next, we explored whether Drs2 is only involved in bulk autophagy or whether it also participates in selective types of autophagy. We therefore turned to the cytoplasm-to-vacuole targeting (Cvt) pathway, a biosynthetic selective type of autophagy in which oligomers formed by precursor Ape1 (prApe1) are delivered from the cytoplasm into the vacuole [4]. In the vacuole, prApe1 is processed into its mature form, i.e. Ape1, and these two Ape1 forms can be distinguished by western blot [4,41]. As expected, prApe1 was processed normally in WT cells under growing conditions, while its maturation was blocked in the *atg1∆* control (Fig. 1C). Unlike earlier studies [44], we found that the lack of Drs2 causes a partial impairment in prApe1 processing, which could be bypassed by nitrogen starvation-induced bulk autophagy (Fig. 1C). The complete bypass required 3 h, reflecting the autophagic flux impairment of *drs2∆* cells. The discrepancy in the Cvt pathway with the previous study [44], might be due to variations in growth conditions and strain backgrounds used as suggested previously concerning phenotype discrepancies in cells lacking *DRS2* [51-53].

Altogether, these measurements show that Drs2 is required for normal progression of bulk and selective autophagy.

### Drs2 is required for autophagosome formation

**Figure 2.**
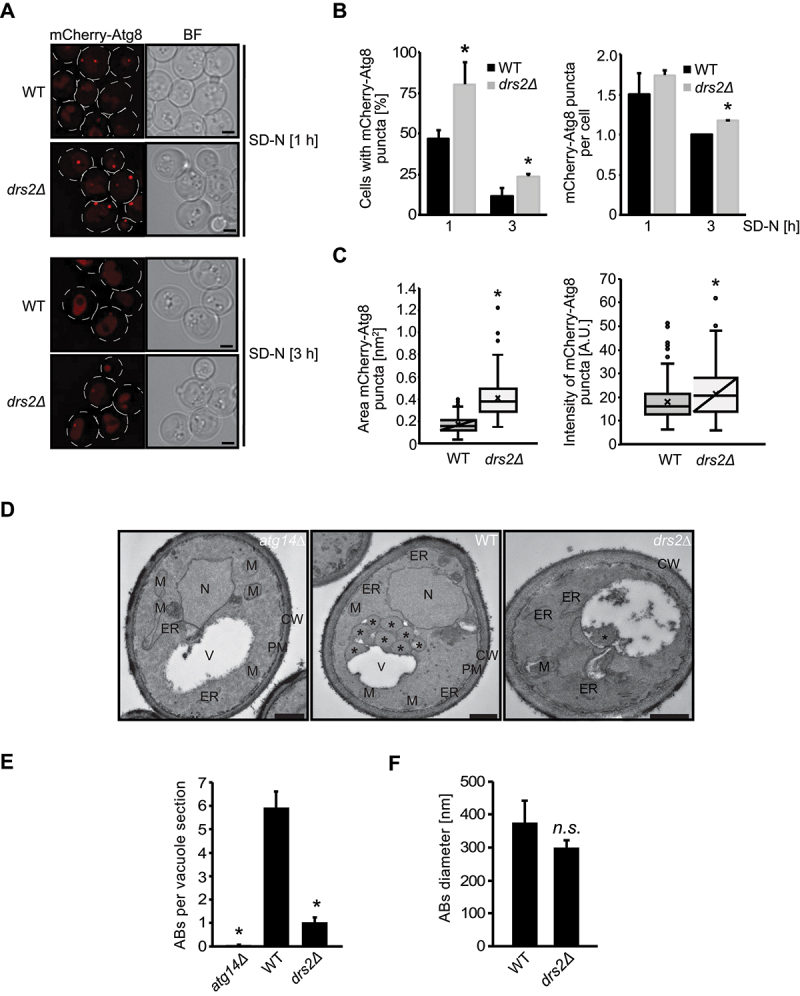
Cells lacking Drs2 have a defect in autophagosome formation. (**A**) WT (FKY826) or *drs2*Δ (FKY827) cells expressing integrated mCherry-Atg8 were grown in SMD medium at 30°C to late log phase and shifted to SD-N medium for 1 or 3 h. Cells were then subjected to fluorescence microscopy. BF, bright field. Size bar: 2 µm. (**B**) The percentage of cells with mCherry-Atg8-positive puncta and their number per cell from (A) was quantified. Bars present the average values from two independent experiments plus SD (n= 2; cells: min. 150). The asterisks indicate significant differences in Atg8 puncta formation between the *drs2*Δ strain and the WT at the indicated time points (two-tailed Student’s t-test, p-value < 0.05 or smaller). (**C**) The fluorescence intensity and pixel area of mCherry-Atg8 puncta after 1 h starvation in SD-N treatment of two experiments was quantified (n=2; cells: 100). The asterisks indicate significant differences in fluorescence intensity and pixel area between the *drs2*Δ strain and the WT at the indicated time points (two-tailed Student’s t-test, p-value < 0.0001). (**D**) The *pep4∆* (WT) (TVY1), the *atg14∆ pep4∆* (*atg14∆*) (RGY553) and the *drs2∆ pep4∆* (*drs2∆*) (FKY831) strains were grown in YPD at 30°C to late log phase and shifted into SD-N for 3 h before being processed for electron microscopy. ER, endoplasmic reticulum; M, mitochondrion; N, nucleus; PM, plasma membrane; V, vacuole. Asterisks highlight ABs. Size bar: 500 nm. (**E**) Quantification of the average number of the ABs per vacuole section in the experiment shown in panel D. The asterisk indicates the significant differences between the *drs2*Δ *pep4*Δ or *atg14*Δ *pep4*Δ cells, and the *pep4*Δ strain (two tailed Student’s t-test; p-value < 0.0001). (**F**) Quantification of the average diameter of the ABs in the experiment depicted in panel D. The abbreviation *n.s*. highlights the non-significant difference between the *drs2*Δ *pep4*Δ and *pep4*Δ strains.

The decreased autophagy levels observed in the *drs2∆* cells could reflect an impairment in either autophagosome biogenesis, autophagosome-vacuole fusion or autophagic body (ABs) degradation. We therefore looked at the subcellular distribution of the mCherry-Atg8 reporter construct by fluorescence microscopy. During autophagy Atg8 localizes to the PAS [7-9]. The presence of a perivacuolar mCherry-Atg8-positive punctum indicates the formation of a late PAS, while the appearance of a dispersed mCherry signal in the vacuolar lumen highlights fusion of autophagosomes with the vacuole and ABs degradation [54]. Using live-cell fluorescence microscopy, we observed both in WT and *drs2∆* cells mCherry-Atg8 puncta formation after 1 h nitrogen starvation, while after 3 h starvation a diffuse mCherry signal accumulated within the vacuolar lumen (Fig. 2A). We noticed, however, that the *drs2∆* mutant showed a 50% increase of cells positive for mCherry-Atg8 puncta compared to WT, without an increase in the number of mCherry-Atg8 puncta in the cytosol or at the vacuole (Fig. 2B). This indicates an impairment and/or retardation in autophagosome formation in *drs2∆* cells compared to WT, while late autophagy steps such as autophagosome fusion with the vacuole are probably not affected, which would otherwise have resulted in an accumulation of mCherry-Atg8 puncta within the cytosol or at the vacuole [54]. We and others previously noticed that in cells defective in autophagosome formation such as *atg1∆* cells [55], fluorescent-tagged Atg8 at the PAS appears as a brighter dot compared to WT. In accordance with a defect early in autophagy, the mCherry-Atg8 puncta in the *drs2∆* mutant appeared enlarged and displayed increased relative fluorescence intensity in comparison to WT (Fig. 2C). Together, these observations suggest that the observed defect in bulk autophagy in the *drs2∆* mutant is caused by reduced and/or delayed autophagosome formation.

To further substantiate the autophagosome formation defect, we examined the number and size of ABs present in the vacuoles of a *drs2∆ pep4∆* mutant strain using electron microscopy. As ABs correspond to the inner vesicle of autophagosomes, which are detectable after autophagosome fusion with the vacuole in the absence of the main vacuolar protease Pep4, the number of these vesicles directly reflect the amount of autophagosomes that can be formed in the cytosol during a precise time period [41]. Whereas there was a pronounced reduction in the number of ABs in the *drs2∆ pep4∆* mutant after 3 h of nitrogen starvation, these ABs were of normal size. Thus, autophagosome formation likely proceeds normally but occurs at decreased rates. Moreover, in agreement with the live-cell imaging results (Fig. 2B), we did not detect complete autophagosomes nor elongated phagophores in the cytoplasm, indicative that late autophagy steps, i.e., closure, maturation and fusion of the autophagosomes, are unaffected.

### Drs2 is involved in Atg9 trafficking

**Figure 3.**
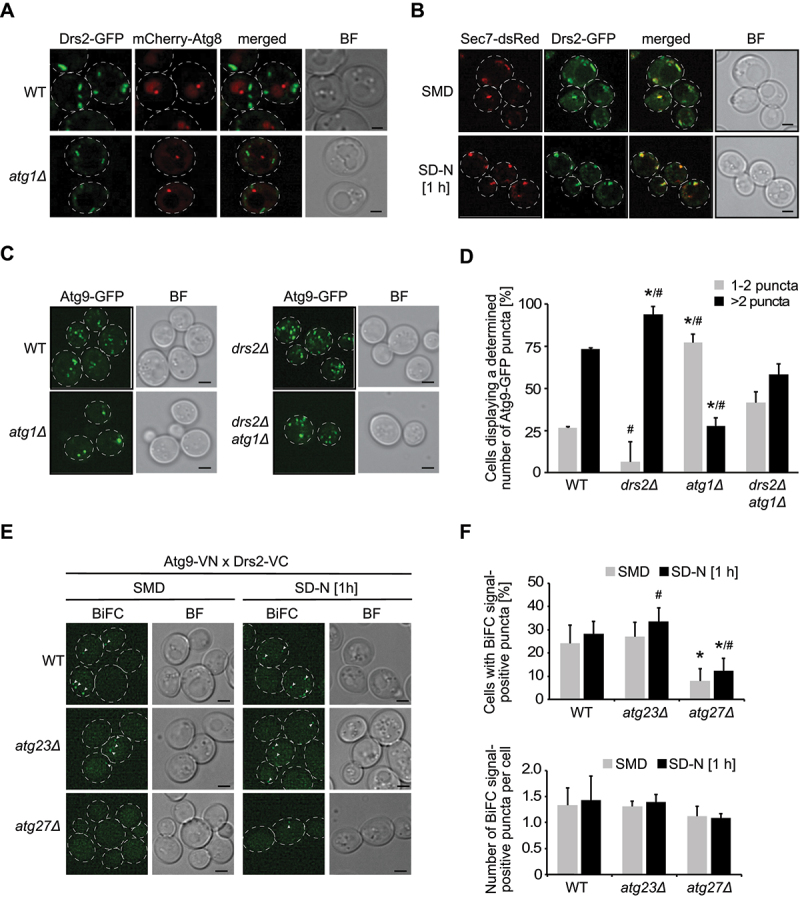
Drs2 regulates Atg9 trafficking at late Golgi compartments. (**A**) Drs2 is not present at the PAS. WT (FKY832) or *atg1*Δ (FKY830) cells expressing both Drs2-GFP and mCherry-Atg8 were grown in SMD to late log phase and shifted to 1 h SD-N before imaging. BF, bright field. Size bar: 2 µm. (**B**) Drs2 largely colocalizes with the late Golgi marker protein Sec7. Cells expressing both Drs2-GFP and Sec7-dsRed (FBY025) were grown in SMD to late log phase and imaged before and after 1 h starvation in SD-N. BF, bright field. Size bar: 2 µm. (**C**) Deletion of *DRS2* causes an Atg9 trafficking defect. The WT (KTY97), *atg1*Δ (SAY001), *drs2*Δ (FKY757) and *drs2*Δ *atg1*Δ (FKY878) strains expressing Atg9-GFP were grown to late log phase and shifted for 1 h to SD-N before imaging. Images are shown as maximum projections from 16 (out of 20) Z-stack slices. BF, bright field. Size bar: 2 µm. (**D**) Quantification of the percentage of cells displaying 1-2 or >2 Atg9-GFP puncta per cell in the experiment shown in (C) (n=2; cells: min 90). Bars present the SD. The asterisks indicate significant differences in Atg9 puncta between WT and *drs2*Δ or *atg1*Δ cells (one-way ANOVA, p-value < 0.05 or smaller). The hashtags indicate significant differences in Atg9 puncta between *drs2*Δ *atg1*Δ and *drs2*Δ or *atg1*Δ cells (one-way ANOVA, p-value < 0.05 or smaller). (**E**) Drs2 and Atg9 are in close proximity. Atg9 and Drs2 were C-terminally tagged with the N-terminal (VN) and C-terminal (VC) split-Venus construct, respectively. Cells expressing both Atg9-VN and Drs2-VC were grown in SMD to late log phase and imaged before and after 1 h starvation in SD-N, in the WT (FKY779), *atg23*Δ (FKY754) or *atg27*Δ (FKY755) background. BF, bright field. Size bar: 2 µm. (**F**) Quantification of both the percentage of cells displaying at least one BiFC signal-positive puncta and the number of BiFC signal-positive puncta per cell from (E) (n=4; cells: min. 100). The asterisks indicate significant differences in the percentage of cells displaying BiFC signal-positive puncta between WT and *atg23*Δ or *atg27*Δ cells (one-way ANOVA, p-value < 0.01). The hashtag indicates significant differences in the percentage of cells displaying at least one BiFC signal-positive puncta under growth and starvation conditions in either the *atg23*Δ or *atg27*Δ mutant (two-tailed Student’s t-test, p-value < 0.05 or smaller).

Drs2 is localized at the TGN, where it flips PS and PE from the lumenal to the cytosolic side of the TGN limiting membrane [28,39,40]. Asymmetric lipid distribution, specifically for PtdIns3P, has also been described in autophagosomal membranes [56]. We were therefore wondering whether Drs2 modulates autophagy at the PAS and explored whether Drs2 associates with mCherry-Atg8 positive puncta. However, we could not detect a clear colocalization between endogenous Drs2-GFP and mCherry-Atg8 (Fig. 3A). Using the *atg1∆* background, which enhances the recruitment of many Atg proteins to the PAS, including Atg8, Drs2 was still not visible at the PAS (Fig. 3A). Hence, we concluded that Drs2 does not redistribute to the PAS under autophagy-inducing conditions. In contrast and as expected [28,42], the Drs2-GFP signal overlapped with the TGN marker protein Sec7 both under growing and nitrogen starvation conditions (Fig. 3B).

Transmembrane Atg9 concentrates in several cytoplasmic tubovesicular reservoirs after being transported to and sorted from the Golgi/TGN [49,57]. From there, Atg9-positive tubovesicular membranes can reach the PAS where they provide part of the membranes required for the formation of the phagophore [18,49,57]. Several Golgi-resident proteins have been implicated in autophagy by impairing Atg9 transport to the PAS [44-50]. To assess Atg9 trafficking to the PAS in cells lacking *DRS2*, we took advantage of the transport of Atg9 after knocking-out *ATG1* (TAKA) assay [54], as also Atg9 pronouncedly accumulates at the PAS in the absence of Atg1 [8,58]. Deletion of genes involved in Atg9 trafficking to the PAS, however, blocks this accumulation of Atg9 in cells lacking *ATG1*. As expected [54,58], endogenous Atg9-GFP was distributed in several puncta throughout the cell in the WT, while it was mainly concentrated in 1-2 puncta per cell in the *atg1∆* deletant (Fig. 3C). Although Atg9-GFP localization in the single *drs2∆* knockout was indistinguishable from that in WT, it was still present in various puncta in about 50% of the *drs2∆ atg1∆* double mutants (Fig. 3D). These results suggest that Drs2 is involved in Atg9 sorting at the TGN and the observed defect in autophagosome formation in *drs2∆* cells is caused by aberrant Atg9 trafficking to the PAS.

Atg9 reaches the PAS from a peripheral pool of highly mobile Atg9-positive tubovesicular membranes that are derived from the TGN [49,57] and, previously Atg9 has been found to colocalize with Drs2 at the TGN when general trafficking from and to the TGN was blocked [57]. We therefore wondered whether Drs2 and Atg9 interact at the TGN. Based on what is known about the transport vesicle formation machinery and cargo proteins, we reasoned that an eventual interaction between Drs2 and Atg9 at the TGN is probably only transient, which makes potential binding difficult to detect. Thus, we decided to monitor proximity between Atg9 and Drs2 by bimolecular fluorescence microscopy (BiFC) [59]. Atg9 and Drs2 were tagged with the N- and C-terminal fragment of Venus, respectively, and in case of close proximity or an association between the two proteins, a stable fluorescent Venus is formed. Since reformation of Venus is partially reversible, BiFC enhances transient colocalizations [59]. In about 25% of the examined cell population, we detected a BiFC signal under growing as well as starvation condition, with an apparent slightly higher fluorescence puncta intensity under nitrogen starvation (Fig. 3E and 3F). This is in agreement with previous proteomics data [17,18], which identified Drs2 as a potentially transient Atg9 interactor and/or a protein being in the same compartment as Atg9. To determine whether the BiFC signal results from an interaction at the TGN rather than at the cytoplasmic Atg9 reservoirs, we depleted this peripheral Atg9 pool by deleting either *ATG23* or *ATG27*, as these genes are essential for the formation of the Atg9-positive compartments [57,60]. Deletion of *ATG23* showed a similar number of the BiFC punctate signals in cells expressing Drs2-VC and Atg9-VN compared to WT, revealing that Drs2 and Atg9 colocalize at the TGN (Fig. 3E and 3F). Interestingly, lack of *ATG27* resulted in a reduction but not an abolishment of the BiFC signal (Fig. 3D and 3E), which is likely due to Atg9 mistargeting to the vacuole in the *atg27∆* deletant [57]. Nonetheless, the data indicate that the interaction between Drs2 and Atg9 occurs at the TGN.

### Drs2 flippase activity is required in autophagy

**Figure 4.**
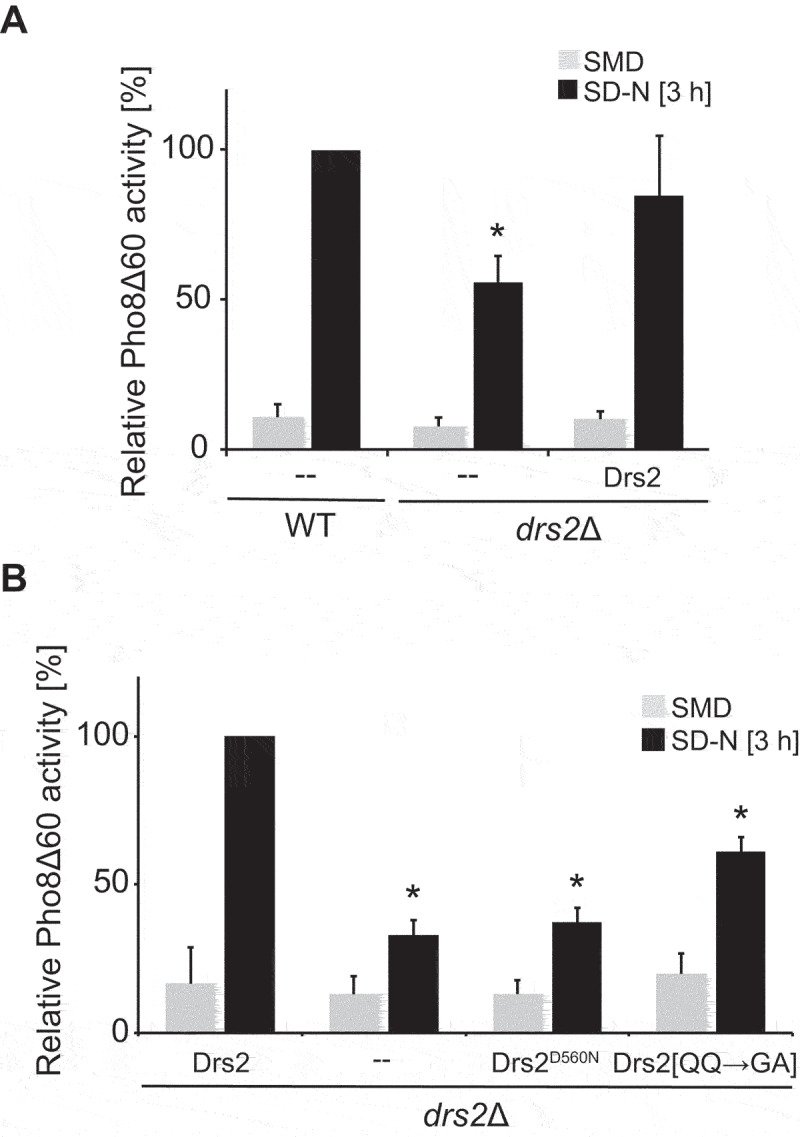
Drs2 flippase activity is important for autophagy. (**A**) C-terminally 13xMYC-tagged Drs2 can complement the autophagy defect of the cells lacking *DRS2*. The *drs2*Δ mutant carrying both the Pho8Δ60 reporter (FBY015) and either an empty vector or a plasmid expressing Drs2-13xMYC was grown in SMD at 30°C to a late log phase and then shifted to SD-N medium for 3 h. Cells were collected before and after the incubation in SD-N, lysed and the Pho8Δ60 activity was measured. All values were normalized the Pho8Δ60 activity of the WT. The bars present the average values from three independent experiments plus SD. The asterisk indicates significant differences in bulk autophagy between WT and *drs2*Δ with empty plasmid at the indicated time point (one-way ANOVA, p-value < 0.01) (**B**) Autophagy depends on the flippase activity of Drs2 and translocation of PE is sufficient to partially sustain autophagy. The *drs2*Δ strain carrying both the Pho8Δ60 reporter (FBY015) and an empty vector or a plasmid expressing either Drs2-13xMYC, the flippase-dead mutant Drs2^D56^^0N^-13xMYC or the PS flippase deficient mutant Drs2[QQ→GA]-13xMYC were grown in SMD-URA at 30°C to late log phase and then shifted to SD-N medium for 3 h. Cells were collected before and after the incubation in SD-N, lysed and the Pho8Δ60 activity was measured. All values were normalized to the Pho8Δ60 activity of the *drs2Δ* strain expressing Drs2-13xMYC. The bars present the average values from three independent experiments plus SD. The asterisk indicates significant differences in bulk autophagy between WT and the differently transformed *drs2*Δ cells at the indicated time point (one-way ANOVA, p-value < 0.0001).

We next wondered whether Atg9 trafficking and hence autophagy depends on the physical presence of the Drs2 protein at the TGN alone, or also on its lipid flippase activity. The lipid flippase activity of Drs2 is ATP dependent and mutation of the catalytic Asp into an Asn at position 560 within the ATPase domain, renders Drs2 flippase inactive [28]. To assess the relevance of Drs2 flippase activity for autophagy progression, we enzymatically measured bulk autophagy in the Pho8∆60 *drs2∆* strain expressing either Drs2-13xMYC or the flippase-dead Drs2^D560N^-13xMYC mutant. Because tagging of Drs2 has been previously described to influence Drs2 activity *in vitro* [31], we first tested whether our Drs2 construct was functional in autophagy using the Pho8∆60 assay. As shown in Fig. 4A, expression of Drs2-13xMYC largely complemented the autophagy defect of *drs2∆* cells. Expression of the ATPase-dead Drs2^D560N^ variant, however, was unable to reverse the autophagy defect of the cells lacking *DRS2* (Fig. 4B). This result shows that the flippase activity of Drs2 is essential for autophagy progression.

Drs2 is known to flip both PS and PE, with a preference for PS [39,40]. PS is an anionic lipid and this class of lipids is known to determine membrane properties such as membrane charge and curvature, and consequently directly influences binding and activity of lipid binding proteins and thus organelle identity [61]. Accordingly, the activity of Drs2 and the consequent exposure of PS at the cytosolic side of the TGN limiting membrane has been shown to be important to specifically recruit trafficking factors to this compartment [51,52]. In particular, the exposure of PS at the cytosolic side of the TGN limiting membrane with its negative charge enhances membrane protein interactions and induces membrane curvature to support vesicle-mediated transport, including AP-1/clathrin-dependent trafficking or endosomal recycling [51-53]. To test whether autophagy also depends on the translocation of PS by Drs2, we took advantage of the previously characterized Drs2[QQ→GA] mutant that is known to abrogate PS flippase activity, while the recognition of PE as a substrate remains intact [23]. Interestingly, impairment of PS translocation could still partially sustain autophagy as cells carrying the Drs2[QQ→GA] mutant show only an intermediate defect in Pho8∆60 activity in comparison to the *drs2∆* mutant expressing WT Drs2 (Fig. 4B). This result is in line with previous observations that showed that PE translocation by Drs2 is also important for intracellular trafficking along the Golgi pathway and in the endosomal system [39,40].

## Discussion

Autophagosome biogenesis is a complex, multistep process that requires the fine orchestration between the action of the Atg machinery and the massive supply of membranes essential for the *de novo* formation of these large vesicles [1,5]. Timely regulated lipid asymmetry within the nascent autophagosome membrane might probably coordinate some of these steps, promoting the association/dissociation of specific proteins and/or changes in the biophysical properties, which could for example induce membrane curvature and lipid packing. Lipid asymmetry could be created through differential lipid biosynthesis and/or modification in one of the lipid bilayers of the phagophore membrane [62]. Other players key in generating membrane asymmetry are lipid flippases, which translocate polar lipids from one lipid layer to the other, in an ATP-dependent manner [19,20]. Their direct relevance in autophagosome biogenesis, however, was unknown. In this study, we systematically examined whether one or more *S. cerevisiae* flippases play a role in autophagy. We found that the aminophospholipid flippases Drs2 and Neo1, which mainly localize to the TGN and various intracellular compartments (ER, Golgi and endosomes), respectively [28,39,40,43,63,64], are important for autophagy progression. Although here we focused on Drs2, future investigations on Neo1 could also shed light on membrane asymmetry and the mechanism of autophagy, especially as the *C. elegans* homologs of the yeast proteins Dop1 and Mon2, which form a complex with Neo1, [63,65], have been shown to participate in autophagy [66].

Our data show that *Drs2* deletion or abrogation of Drs2 flippase activity lead to an autophagy impairment. The block is not complete but this could be explained by the fact that Drs2 is partially redundant with Dnf1, Dnf2 and Dnf3 [38], and therefore the absence of Drs2 can probably be compensated to a certain extent by the activity of these three flippases. Since Drs2 is involved in protein sorting at the TGN, *Drs2* deletion affects the secretion and the trafficking within the endosomal system of numerous proteins [28,38,67]. Consequently, the observed autophagy defect could be indirect. However, we found that Drs2 is specifically involved in the anterograde transport of Atg9 to the PAS, which is consistent with our detection of proximity between Atg9 and Drs2 by BiFC. Moreover, proteomics analyses done by others in the presence of a crosslinker and a mild detergent, or upon microsome isolation, indicated that Drs2 could be associated with Atg9 and/or present in the same compartment as Atg9, as well as Atg23 [17,18]. Atg23 is a protein that is in complex with Atg9 [68,69]. Atg9 levels and consequently its abundance at the PAS determines the frequency of autophagosome formation but not the size of these vesicles [70]. Our results are also consistent with this notion. That is, Atg9 delivery to the PAS is impaired in *drs2∆* cells and as highlighted by our electron microscopy analysis, the generated autophagosomes have normal size but in reduced number. Thus, the autophagic flux defect of the *drs2∆* mutant is due to a reduction in the autophagosome formation frequency.

Previously, several factors that participate in protein sorting from the TGN, including the GTPases Arf1, Arf2, Sec4, the GEFs Sec2, Sec7, Gea1 and Gea2, and the phosphatidylinositol 4-kinase Pik1, have been found to play a role in both selective and bulk autophagy [44-48,50,71]. Interestingly, most of them are crucial for Atg9 delivery to the PAS [44-48,50,71]. It has been shown that Drs2 directly cooperates with some of those factors, such as phosphatidyl-inositol-4-phosphate (solely generated by Pik1 in yeast), Arl1 and Gea2, in the sorting of cargo proteins at the TGN [30,32,72,73]. This could explain the phenotype similarities in autophagy and Atg9 anterograde transport to the PAS defect in cells lacking *DRS2* and the strains deficient in the above-mentioned factors. Thus, a very likely scenario is that Drs2 and the above-mentioned proteins are important for Atg9 sorting from the TGN and the subsequent formation of the tubovesicular Atg9-positive membranes that act as a precursor structure for the PAS upon its reposition adjacent to both the vacuole and the ER [18,49,57]

In conclusion, our study has uncovered Drs2 as a novel autophagy player, which specifically regulates anterograde Atg9 transport to the PAS. Since Atg9 delivery to the PAS is epistatic to most of the other steps of autophagosome biogenesis [49,57], we cannot exclude that aminophospholipid asymmetry generated by this flippase is also important for other steps of autophagy in which the Atg9-positive membranes are involved. Future investigations will be important to address these aspects of the mechanism of autophagosome formation and eventually identify the functional counterpart(s) of Drs2 in mammalian cells.

## Material and Methods

### Strains and media

*S. cerevisiae* strains used in this study are listed in Table 1. Genes were deleted via homologous recombination using PCR fragments that contain a marker cassette flanked by approximately 60 bases identical to the regions flanking the targeted open reading frame [74,75]. Gene knockouts were verified by PCR. C-terminal tagging was achieved by PCR-based integration at the 3’ end of the chromosomal locus of the targeted gene. Expression of tagged proteins was verified by live-cell imaging and/or western blot.

**Table 1.** Strains used in this study.

Strain	Genotype	Source
BY4727	*MATα his3Δ200 leu2Δ0 lys2Δ0 met15Δ0 trp1Δ63 ura3Δ0*	EUROSCARF
FBY015	SEY6210 *pho13Δ::KAN pho8::PHO8Δ60 drs2Δ::HPH*	This study
FBY025	SEY6210 *DRS2-GFP:NAT pRS406Sec7-6xdsRED*	This study
FKY754	BY4727 *ATG9-VN::HIS3 DRS2-VC::TRP1 atg23Δ::HPH*	This study
FKY755	BY4727 *ATG9-VN::HIS3 DRS2-VC::TRP1 atg27Δ::NAT*	This study
FKY757	SEY6210 *ATG9-GFP::TRP1 drs2Δ::HPH*	This study
FKY758	SEY6210 *drs2Δ::HPH*	This study
FKY779	BY4727 *ATG9-VN::HIS3 DRS2-VC::TRP1*	This study
FKY826	SEY6210 *pRS406Cupr-mCherryV5-Atg8::URA3*	This study
FKY827	SEY6210 *drs2Δ::HPH pRS406Cupr-mCherryV5-Atg8::URA3*	This study
FKY830	SEY6210 *DRS2-GFP:NAT atg1Δ::TRP1* *pRS406Cupr-mCherryV5-Atg8::URA3*	This study
FKY831	SEY6210 *drs2Δ::HPH pep4Δ::LEU2*	This study
FKY832	SEY6210 *DRS2-GFP:NAT* *pRS406Cupr-mCherryV5-Atg8::URA3*	This study
FKY878	SEY6210 *ATG9-GFP::TRP1 atg1Δ::URA3 drs2Δ::HPH*	This study
KTY97	SEY6210 *ATG9-GFP::TRP1*	[58]
RGY553	SEY6210 *atg14Δ::hphNT1 pep4Δ::HIS5 S.p.LoxP*	Reggiori lab collection
SEY6210	MAT*α ura3-52 leu2-3,112 his3-Δ200 trp1-Δ901 lys2-801 suc2-Δ9 mel GAL*	[82]
SAY001	SEY6210 *ATG9-GFP::TRP1 atg1Δ::URA3*	[79]
TPY010	SEY6210 *drs2Δ::HIS3LoxP*	J. Holthuis
TPY015	SEY6210 *dnf3Δ::HIS3LoxP*	J. Holthuis
TPY026	SEY6210 *dnf1Δ::LoxP dnf2Δ::HIS3LoxP*	J. Holthuis
TVY1	SEY6210 *pep4Δ::LEU2*	[83]
WHY1	SEY6210 *atg1Δ::HIS5 S.p.*	[84]
ZHY124-34A2B	Mat*α* his3 leu2 ura3 ade2 trp1 suc2 *neo1-2::HIS3-KanMx*	[43]
YTS159	SEY6210 *pho13Δ::KAN pho8::PHO8Δ60*	[85]

Yeast cells were grown in rich medium (YPD; 1% yeast extract, 2% peptone, 2% glucose) or synthetic minimal media (SMD; 0.67% yeast nitrogen base, 2% glucose, and amino acids and vitamins as needed) at permissive temperature of 30°C or 23°C unless otherwise indicated to a late logarithmic phase of 0.8-1.2 OD_600_. Starvation was induced by shifting the cells to synthetic media lacking nitrogen (SD-N; 0.17% yeast nitrogen base without amino acids, 2% glucose).

## Plasmids

The following plasmids pRS404 [76], pRS416 [76], pRS416CuprGFP-Atg8 [77], pRS406mCherryV5-Atg8 [49], pSSEC7DsRed.M1x6 [78] have been described elsewhere. Although the expression of the Atg8 fusions is driven by the *CUP1* promoter, no extra cupper was added since the level already present in the media is sufficient to lead to a slight overproduction of the chimeras. The Drs2-13xMYC and Drs2^D56^^0N^-13xMYC plasmid was created by amplifying the WT and the ATPase-dead *DRS2* ORF from the previously published pRS426_3xHA10xHisDrs2 and pRS426_3xHA10xHisDrs2^D56^^0N^ plasmid [35], respectively, and cloned into pRS404 using EcoRI and XmaI. The *DRS2* gene was placed under the control of its endogenous promotor, 576 bp before the start codon, amplified from genomic DNA and cloned before the *DRS2* ORF using SalI and EcoRI. The 13xMYC tag and the *ADH1* terminator were excised from pRS416 Atg4-13xMYC [79] using XmaI and SacI, and cloned at the 3’ end of *DRS2*. This procedure led to the creation of the pRS404Drs2-13xMYC and pRS404Drs2^D56^^0N^-13xMYC plasmids. To create the Drs2[QQ→GA] mutant, a DNA fragment carrying the mutation was obtain via overlap extension PCR on *DRS2* with the mutation present in the overlapping primers. The generated fragment was then cloned into pRS404Drs2-13xMYC plasmid using AgeI and PstI to replace the WT region and the insertion of the correct mutation was verified by DNA sequencing. This generated pRS404Drs2[QQ→GA]-13xMYC. Each construct including the *DRS2* promoter, *DRS2* ORF, 13xMYC-tag and the *ADH1* terminator, was transferred from pRS404 into pRS416 using SalI and SacI, to create the pRS416Drs2-13xMYC, pRS416Drs2^D560^^N^-13xMYC and pRS416Drs2[QQ→GA]-13xMYC plasmids.

## Biochemical assays

Pho8∆60 activity measurement was performed as previously described [41]. In brief, the indicated strains were pre-cultured overnight in YPD at 30°C, while the temperature sensitive strain *neo1-2* was grown at 23°C. Cells were diluted in YPD and allowed to undergo 2-3 divisions before harvesting. Five OD_600_ equivalent of cells were mechanically lysed in 20 mM PIPES, pH 6.8, 100 mM KAc, 50 mM KCl, 10 mM MgCl_2_, 10 µm ZnSO_4_, 0.5% Triton X-100 (Sigma-Aldrich, 93443-100ML), 1 mM PMSF (Sigma-Aldrich, P7626-5G). Cell debris were removed by centrifugation at 16,000 x g for 5 min. The pre-cleared cell extract was mixed with the ALP reaction buffer (250 mM Tris-HCl, pH 8.5, 10 mM MgCl_2_, 10 µm ZnSO_4_, 0.4% Triton X-100, 1.25 mM p-nitrophenyl phosphate [Sigma-Aldrich, N2765-100TAB]) in a ratio of 1:5. The colorimetric change of the p-nitrophenyl phosphate was measured at 37°C every 3 min for 20 min using a GloMax-Multi Detection System (Promega). The activity of Pho8∆60 was determined by plotting the absorbance measurements against the time. The obtained slopes were normalized to the protein concentration of the cell extracts, which were measured using the Pierce^TM^ BCA Protein Assay Kit (ThermoFisher Scientific, 23227) also using the GloMax-Multi Detection System. The ALP activity of WT cells after starvation was set to 100%.

The analysis by western blot of GFP-Atg8 or prApe1 processing was performed as described previously [41]. Cells were diluted in YPD and allowed to undergo 2-3 division cycle before harvesting. Two OD_600_ units of cells were precipitated in 10% trichloroacetic acid (TCA), acetone washed and subjected to SDS-PAGE electrophoresis followed by western blotting using anti-GFP (Roche, 11814460001) or anti-Ape1 antibody [49], respectively.

## Western blotting

In general, 2 OD_600_ equivalent of cells were precipitated in 10% TCA, acetone washed and subjected to SDS-PAGE electrophoresis followed by western blot using as the primary antibodies the monoclonal anti-GFP or rabbit anti-Ape1, and as the secondary antibodies Alexa Fluor 680-conjugated goat anti-rabbit or anti-mouse IgG (ThermoFisher Scientific, A-21109 and A-21058, respectively). Detection was performed using an Odyssey® Fc Imaging System (LI-COR Biosciences).

## Fluorescence microscopy and image analysis

Cells were pre-cultured in SMD medium overnight, diluted in SMD medium, grown for 2-3 division cycles and transferred to SD-N medium for the indicated times. Cells were briefly washed with water and images were acquired with a DeltaVision Elite RT microscope system (GE Healthcare, Applied Precision), equipped with a UPLASPO 100× oil/1.40 NA objective, a pco.edge 5.5 sCMOS camera (PCO) and a seven-color InsightSSI solid-state illumination system (GE Healthcare, Applied Precision). Z-stacks of 15 or 20 focal planes 0.2 µm apart were collected for each fluorescent channel. BiFC images were taken by collecting a Z-stack of 3 focal planes 0.4 µm apart to reduce bleaching. Images were then deconvolved using the SoftWoRx software (Applied Precision). BiFC signal and Atg8 puncta quantification was performed by manual counting using Fiji [80] or ImageJ (National Institutes of Health, Bethesda, MD), respectively. The mean fluorescent intensity and pixel area of Atg8 puncta was measured using ImageJ.

## Electron microscopy

Electron microscopy examinations were performed and quantified as previously described [81]. The indicated strains were pre-cultured overnight in YPD at 30°C, diluted in YPD and grown through 2-3 division cycles. Cells were then shifted to SD-N for 3 h, harvested, briefly washed in water and resuspended and incubated in ice-cold 1.5% KMnO_4_ (Sigma Aldrich, 223468-25G) for 30 min at 4°C on a rotatory wheel. Cells were centrifuged at 3,000 x g for 3 min at 4°C and the resuspended in 1.5% KMnO_4_ for an incubation overnight at 4°C. After 5 brief washes in water, cells were dehydrated progressively in a gradient of 10, 30, 50, 70, 90 and 95% acetone and three times with water-free acetone, with at least a 20-min incubation for each step on a rotatory wheel at room temperature. Subsequently, cells were incubated in 33% Spurr’s resin (911.8 g nonenyl succinic anhydride [Sigma-Aldrich, 74378-250ML], 8.2 g ERL 4221 epoxide [Ted Pella, 18306-4221], 1.9 g diglycidyl ether of polypropyleneglycol 736 [Sigma-Aldrich, 31191-250G], 0.2 g dimethylaminoethanol [Ted Pella,18315]) in acetone for at least 1 h at room temperature and then in 100% Spurr’s resin overnight. Cells were finally transferred to conic embedding capsules (BEEM embedding capsules size 00; Electron Microscopy Sciences, 70010-B) and Spurr’s resin to polymerize at 65°C for 4 days.

Ultra-thin 55-nm sections were cut using a Leica ultramicrotome (Leica Microsystems) and collected on formvar carbon-coated 50-mesh copper grids (EMS, G50-Cu). Cell sections were stained with lead-citrate solution (80 mM lead nitrate, 120 mM sodium citrate, pH 12) and analysed in a CM100bio TEM (ThermoFisher Scientific). The average number of ABs per cell section was determined by randomly counting a total of 100 cells in 3 different grids for each condition. The average diameter of ABs was measured using the Photoshop software (Adobe).

## Statistical analyses

Data represent the average of minimum two independent biological replicates ± standard deviation (SD). Statistical significance is indicated within the graph by an asterisk or hashtag. Significance was calculated using two-tailed Student’s t-test pairwise comparison or one-way ANOVA with Dunnett’s multiple comparison test for group comparison. Quantification of the western blot signals was carried out using the Fiji software.
